# Early Intervention Combined with Targeted Treatment Promotes Cognitive and Behavioral Improvements in Young Children with Fragile X Syndrome

**DOI:** 10.1155/2012/280813

**Published:** 2012-03-26

**Authors:** Tri Indah Winarni, Andrea Schneider, Mariya Borodyanskara, Randi J. Hagerman

**Affiliations:** ^1^Medical Investigation of Neurodevelopmental Disorders (MIND) Institute, University of California at Davis Medical Center, Sacramento, CA 95817, USA; ^2^Department of Pediatrics, University of California at Davis Medical Center, Sacramento, CA 95817, USA; ^3^Center for Biomedical Research, Faculty of Medicine, Diponegoro University, Jl. Dr. Soetomo No. 14, Central Java, Semarang 50231, Indonesia; ^4^College of Osteopathic Medicine, Touro University California, 1310 Johnson Lane, Mare Island, Vallejo, CA 94592, USA

## Abstract

Fragile X syndrome (FXS) is the most common inherited cause of intellectual disability due to an expansion in the full mutation range (>200 CGG repeats) of the promoter region of the *FMR1* gene leading to gene silencing. Lack of FMRP, a critical protein for dendritic spine formation and maturation, will cause FXS. Early environmental enrichment combined with pharmacological intervention has been proven to rescue dendritic spine abnormalities in the animal model of FXS. Here we report on 2 young children with FXS who were treated early with a combination of targeted treatment and intensive educational interventions leading to improvement in their cognition and behavior and a normal IQ.

## 1. Introduction

Fragile X syndrome (FXS) is caused by a CGG trinucleotide repeat expansion located in the 5′ untranslated region (UTR) of fragile X mental retardation 1 (*FMR1*) gene. When the CGG expansion is >200 repeats, there is silencing of transcription through methylation and a subsequent deficit or absence of fragile X mental retardation protein (FMRP), the protein product of *FMR1* gene. FMRP is an RNA-binding protein that transports and stabilizes messenger ribonucleic acids (mRNAs) to the synapse, where it regulates protein synthesis usually through suppression of translation [1, 2]. The allele frequency of the full mutation is about 1 in 2500 in the general population [3].

The lack of FMRP leads to a combination of cognitive impairments, behavioral problems, and physical features, including prominent ears and hyperextensible finger joints that are the phenotype of FXS [4]. There are the behavioral and developmental problems in childhood and a variety of medications have been helpful for treating anxiety, aggression, attention deficit hyperactivity disorders (ADHDs), and mood instability [5].

The absence of FMRP causes impairment of synaptic plasticity [6] that includes upregulation of various proteins including matrix metalloproteinase 9 (MMP9) [7], upregulation of the metabotropic glutamate receptor 5 (mGluR5) pathway [8], and downregulation of the gamma-aminobutyric acid (GABA) receptors [9, 10]. The development of targeted treatments for FXS has led to trials of minocycline [11, 12], Arbaclofen [13] and mGluR5 antagonists [14, 15] in patients with FXS. 

Normal intelligence quotient (IQ) is uncommon in males with FXS (13%) and these individuals typically have an unmethylation fully expanded *FMR1* gene mutation [16]. Approximately from 30 to 50% of females with FXS can have a normal IQ [17, 18], and these individual have a favorable activation ratio and higher FMRP level [19]. Here we present 2 cases of children with the FXS who have had a remarkable response to combined interventions and a normal IQ.

## 2. Case Report

Case 1 is a 3-year-old boy carrying a full mutation allele with 245, 310, 523, 723, 1030, and 1360 CGG repeats, and 90% of his *FMR1* alleles are methylated. Mother had normal pregnancy and delivered full term by C-section due to preeclampsia, and he was 3700 grams at birth and suffered from torticollis and frequent emesis in the first year of life. His developmental milestones were delayed with sitting at 7-8 months, crawling at 14 months, walking independently at 18 months, and saying single words at 2.5 years and short sentences at 3 years. He has behavior problems including severe tactile defensiveness, poor eye contact, hyperactivity, and intermittent aggression. At age 2 years and 6 months he started sertraline at 2.5 mg/day with a subsequent dramatic improvement in expressive language described as an “explosion of verbalizations” and the onset of phrases in speech. Minocycline (12.5 mg/day) was started two months before he turned three years, and improvement was seen in his anxiety and aggression. Minocycline was discontinued after 4-month treatment, resulting in the return of his behavior problems including frequent biting, chewing on his shirt, and an increase in his anxiety, so it was restarted. In conjunction with medication, he received 30 hours of physical therapy during his first year of life. After age 2 he received 1 hour of occupational therapy monthly. Now, he attends a day care program for children with special needs for 3 hours/day, 3 days per week. Additionally, his mother, who is a teacher at a vocational school, applies elements of Montessori homeschooling into his daily routine. The educational intervention emphasizes age-appropriate games and exercises in order to promote cognition and memory, concentration, speech, and fine and gross motor skills (e.g., singing, looking at books, applying Montessori materials, etc.).

Examination at age 3 years and 2 months demonstrated normal growth percentiles, hyperextensible finger joints, and severe flat feet. On the Stanford-Binet his full-scale IQ was 94 with a nonverbal of 97 and a verbal of 92, fluid reasoning was 97, overall knowledge 111, quantitative reasoning 89, visual spatial abilities 94, and working memory 86. On the Autism Diagnostic Observation Scale (ADOS), he scored in the normal range. On the Vineland Adaptive Behavior (VAB) Scales, his communication score was 85, daily living skills 91, socialization 79, and overall adaptive behavior composite 86. His motor composite on the McCarthy Scales of Children's Ability was in the average range with a scale index of 54.

Case 2 is the sister of case 1, 7 years 11 months with a full mutation allele (260–370 CGG repeats), and an activation ratio was 0.2 (only 20% of her calls have the normal X as the active X) in peripheral blood lymphocytes. Her mother's pregnancy was complicated by preeclampsia in the last week, and she was delivered by C-section; birth weight was 2980 grams. She was hypotonic, and her developmental milestones were mildly delayed, sitting at 10 months, crawling at 12 months, walking at 16 months, saying words at 20 months, and putting together phrases at 3 years of age. However, her behavioral and emotional problems have been significantly problematic. When she was 20 months, she intermittently refused to walk and she had episodes of selective mutism. She had anxiety on a daily basis, poor eye contact, moodiness, tactile defensiveness, perseveration, and shyness with impaired social interactions. She is in the second grade attending regular school, but she demonstrates learning disabilities in math, reading, and writing.

Psychopharmacological intervention included memantine 5 mg/day started at age 7 with remarkable improvement in reading, writing, and behavior. Three months later, 20 mg/day of sertraline was added which improved her anxiety. Seven months later, minocycline (12.5 mg/day) was added, and it was helpful for math academically. Minocycline was discontinued during the summer holiday, and her math abilities rapidly deteriorated, so it was restarted. She also receives an intensive educational support program with elements of Montessori homeschooling. Math exercises are visualized, and her mother promotes social skills by planning social activities regularly including outside events (theater, horseback riding) and inviting schoolmates to the home with prepared activities, encouraging phone calls with familiar people, and running errands to the supermarket independently built self-confident in socializing.

At 7 years 11 months of age, with cognitive testing utilizing the Stanford-Binet, her full-scale IQ was 98, nonverbal was 87, verbal was 105, fluid reasoning is 94, knowledge is 100, quantitative reasoning is 92, visual spatial abilities 100, and working memory is 97. On the ADOS her score was 4 and in the normal range. On the Vineland, her communication score was 100, daily living skills was 83, socialization was 83, and overall adaptive behavior composite was 87.

## 3. Discussion

 We describe here two young children with FXS who have a normal IQ. They have received intensive educational intervention since early childhood by the mother, a teacher. In addition they have had off-label psychopharmacological interventions. Prior to the combined treatment, both of the children have a developmental history suggesting significant problems related to the full mutation. Case 2 has a low AR of 0.2, and this would be associated with a lower IQ within the broad range of involvement in females [19]. Only 13% of boys with a full mutation have an IQ >70 [16], and an IQ of 94 is very rare [4]; moreover he has 90% methylated alleles which suggests that his IQ would not be high. However, these children demonstrated remarkable behavioral changes and learning ability on the combined medical and educational interventions they received. It is likely that their intensive intervention had a positive effect on their cognitive ability.

 Memantine, an N-methyl-D-aspartic acid (NMDA) noncompetitive receptor antagonist, is a neuroprotective agent that blocks glutamate toxicity and restores homeostasis in the glutamatergic system [20], and this system is upregulated in FXS. The NMDA receptor has been involved in the pathophysiology of autism, and studies have reported improvement of disruptive and aggressive behavior in autism spectrum disorders (ASDs) with memantine [21–23]. Currently, memantine is undergoing controlled trials in autism [21], and open-label in FXS with comorbid ASD had been done showing efficacy [24].

Sertraline is a selective serotonin reuptake inhibitor (SSRI) used with demonstrated efficacy in depression, anxiety, and obsessive compulsive disorders (OCDs) [25]. Low doses of sertraline have minimal adverse effects compared to other SSRIs with less activation and reported benefit in children [5, 26]. SSRIs have been known to stimulate neurogenesis [27, 28]. A recent report of the use of another SSRI, fluoxetine, in the mouse model of Down syndrome demonstrated enhanced neurogenesis when used after birth with increased levels of brain-derived neurotropic factor (BDNF) and enhancement in cognition [29]. BDNF will rescue the synaptic deficits of FXS [30]. Previous studies suggested improvements in behavior and language with the use of SSRIs in younger children with FXS or autism [31, 32]. Approximately 70% of individuals with FXS respond well to fluoxetine with improvements in anxiety and aggression in one open-label study, but hyperarousal was seen in 25% [33].

The third medication utilized is minocycline which lowers MMP9 levels which are too high in FXS. Minocycline leads to dendritic spine maturation both in culture and *in vivo FMR1* knock-out (KO) mice and improves behavior and cognition [7]. The beneficial effect of minocycline has been reported in approximately 70% in a survey of 50 patients with FXS especially in language and behavior [12]. An open-label study of 20 individuals with FXS who were 13–32 years old treated for 8 weeks with minocycline demonstrated improvements with minor side effect [11]. A recent study demonstrated that minocycline significantly potentiated nerve-growth-factor- (NGF-) induced neurite outgrowth in PC12 cells [34]. Moreover, a marked increase of the eukaryotic translation initiation factor (eIF4A1) protein is induced by minocycline, which may be involved in the active mechanism for NGF-induced neurite outgrowth [34].

Excellent enrichment of the environment by parents is another important aspect of their treatment. Spike-timing-dependent long-term potentiation (tLTP) in the prefrontal cortex, which is involved in higher cognitive function, was restored to wild-type (WT) level by an environmental enrichment in the *Fmr1* KO mouse [35]. In another study of the *fmr1* KO mouse an enriched environment rescued the abnormalities of the dendritic spines [36].

Overall the combination of intensive education and the incremental effect of three off-label medications had remarkable effect on behavior and cognition in these children [5]. Although background genetic effects may have had an important influence, their initial significant phenotypic involvement with FXS followed by improvements with combined therapy is noteworthy in these cases. These histories suggest that aggressive early treatment in FXS can lead to an excellent outcome.
